# Supplementary figures and images for: BootCellNet, a resampling-based procedure, promotes unsupervised identification of cell populations via robust inference of gene regulatory networks
Source: PLoS Comput Biol. 2024 Sep 30;20(9):e1012480. doi: 10.1371/journal.pcbi.1012480 (PMC11466406; doi:10.1371/journal.pcbi.1012480)

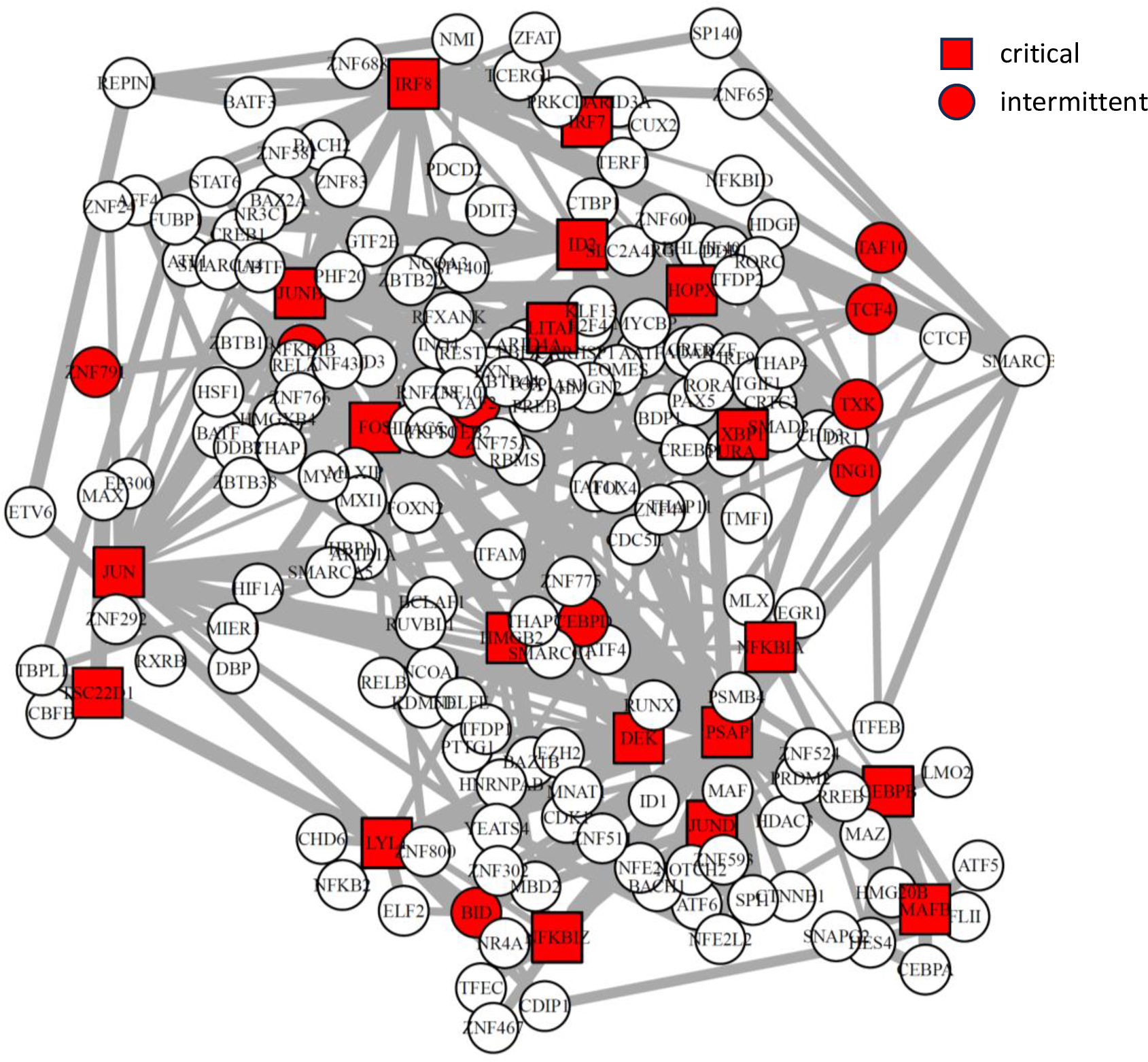

Supplement: S1 Fig — (TIF) [file pcbi.1012480.s001.tif]

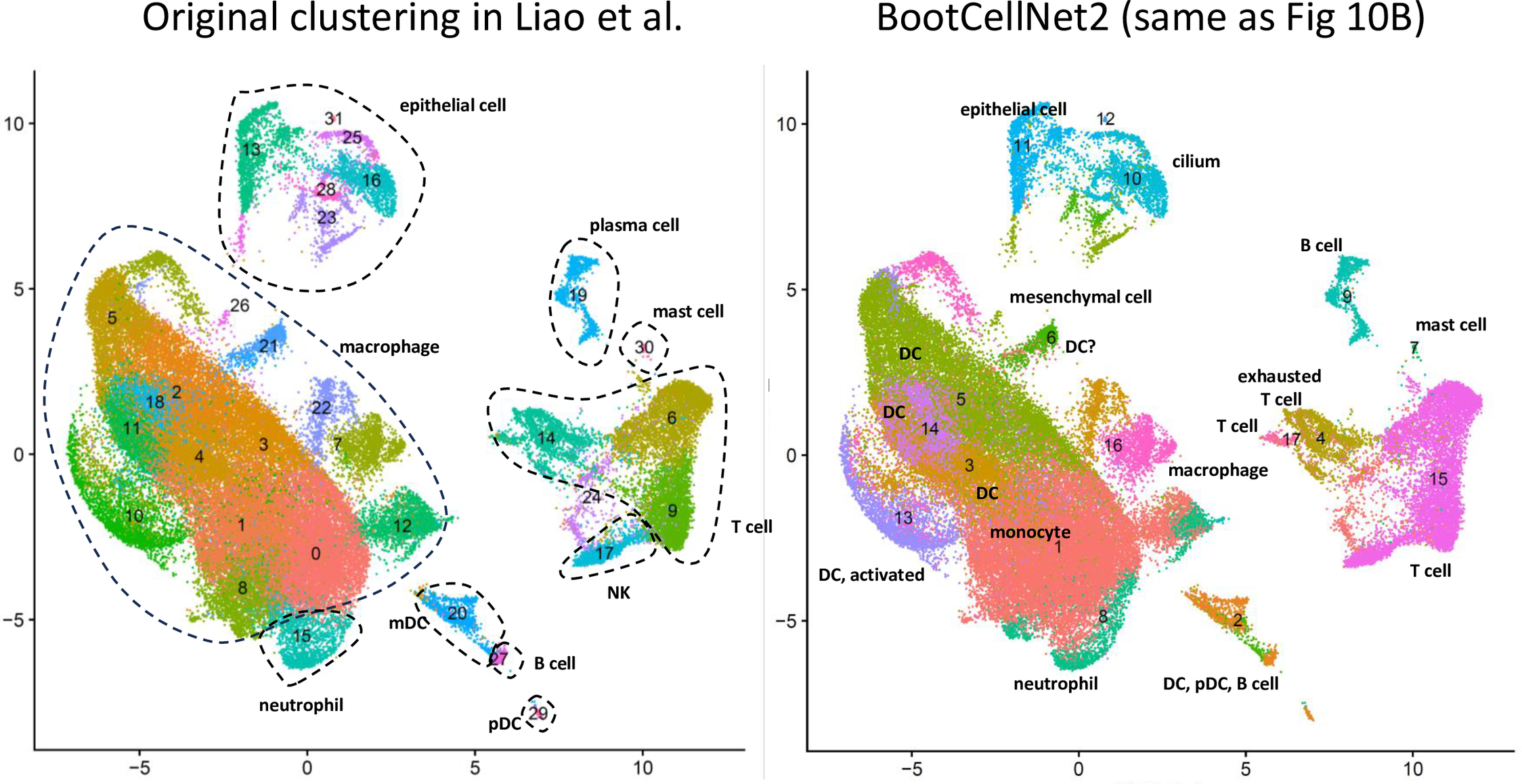

Supplement: S2 Fig — The result was reproduced from the deposited data and is shown (left). Cell type labeling based on the original paper is shown. As a comparison, the MDS-based clustering (same as Fig 9B) is also shown. (TIF) [file pcbi.1012480.s002.tif]
