# Supplementary material for: BootCellNet, a resampling-based procedure, promotes unsupervised identification of cell populations via robust inference of gene regulatory networks
Source: PLoS Comput Biol. 2024 Sep 30;20(9):e1012480. doi: 10.1371/journal.pcbi.1012480 (PMC11466406; doi:10.1371/journal.pcbi.1012480)
Supplement: Attachment — Submitted filename: 20240808_1_BCN_R2R.pdf [file pcbi.1012480.s017.pdf]

Thank you very much for submitting your manuscript "BootCellNet, a resampling-based procedure, promotes unsupervised identification of cell populations via robust inference of gene regulatory networks." for consideration at PLOS Computational Biology.

As with all papers reviewed by the journal, your manuscript was reviewed by members of the editorial board and by several independent reviewers. In light of the reviews (below this email), we would like to invite the resubmission of a significantly-revised version that takes into account the reviewers' comments.

In particular, as requested by Reviewer 2, pay particular attention to the reproducibility of your analysis, to the sharing of all codes and data required in this respect, and to make the corresponding tutorial more easy to follow.

We cannot make any decision about publication until we have seen the revised manuscript and your response to the reviewers' comments. Your revised manuscript will be sent to reviewers for further evaluation.

When you are ready to resubmit, please upload the following:

[1] A letter containing a detailed list of your responses to the review comments and a description of the changes you have made in the manuscript. Please note while forming your response, if your article is accepted, you may have the opportunity to make the peer review history publicly available. The record will include editor decision letters (with reviews) and your responses to reviewer comments. If eligible, we will contact you to opt in or out.

[2] Two versions of the revised manuscript: one with either highlights or tracked changes denoting where the text has been changed; the other a clean version (uploaded as the manuscript file).

Important additional instructions are given below your reviewer comments.

Please prepare and submit your revised manuscript within 60 days. If you anticipate any delay, please let us know the expected resubmission date by replying to this email. Please note that revised manuscripts received after the 60-day due date may require evaluation and peer review similar to newly submitted manuscripts.

Thank you again for your submission. We hope that our editorial process has been constructive so far, and we welcome your feedback at any time. Please don't hesitate to contact us if you have any questions or comments.

Sincerely,

Denis Thieffry, PhD
Academic Editor
PLOS Computational Biology

Pedro Mendes
Section Editor
PLOS Computational Biology

****

Dear Editors and Reviewers,

Thank you for giving me the opportunity to revise my paper. The comments were quite constructive and I believe the changes according to them strengthened the work. Here are the major changes made:

(1) Two comments from reviewer 1 and reviewer 2 prompted me to automate cluster number identification. As I discussed the idea briefly in the Discussion section of the original manuscript, stable clusters may be detected by using pvclust R package. This enables us to (semi-) automatically determine the number of clusters once we give a cutoff p value. I analyzed the stability of the automation step as in Figure 7. The result indicates that the new automated procedure offers more robust clustering than the old procedure.

(2) I also implemented the calculation of critical and intermittent nodes, as suggested by reviewer 3. This was also discussed in the Discussion section of the original manuscript briefly. The critical nodes are defined as nodes included in all the MDS, while the intermittent ones are those included in not all but at least once. Using the union of critical and intermittent nodes, we could robustly cluster the cell clumps as shown in Figure 7.

(3) I made it clear that the paper is a methodology paper. First, I focused on comparison with unsupervised clustering (normal Louvain algorithm) to check the obtained clusters were consistent with immunological knowledge. As reviewer 2 pointed out, the labeling of cells was expert-based (namely, it was I who labeled the cell clusters using my knowledge of immunology). I stated this clearly, by writing the organization of the paper in the Introduction.

(4) I added some discussions on the comparison of our results to unsupervised clustering to strengthen the validation of the results. In particular, we utilized CITE-seq data to validate clustering results as shown in Figure 9.
The primary intention of the analysis presented in the paper was to demonstrate the robustness of our method, particularly given the probabilistic steps involved and the presence of noise within the data. As reviewers pointed out, scRNA-seq data analysis includes a variety of tools for clustering. However, direct comparison and validation are challenging due to the lack of a universal 'ground truth' in clustering and cell type labeling in scRNA-seq data. I believe that the demonstration of the statistical robustness provided by our resampling and automated cluster determination methods, combined with expert-based biological validation, is the best we can offer.

Response to Reviewer #1

Reviewer #1: The work presents a novel method for inference of gene regulatory networks and cell types from single-cell RNA sequencing. The paper relies, among other things, on unsupervised machine-learning techniques such as clustering. However, it solves two major challenges with that kind of approach: inferring the number of clusters and reporting interpretable results.

The authors compare their unsupervised methods with a supervised procedure in Figure 5. However, they mention that there are also unsupervised methods in the literature. They should compare their approach with these, too. As a suggestion, they could contrast their results in the section on hematopoiesis and COVID-19 with the results found by other methods. This would make those sections more pleasant to read. The authors can skip this suggestion, but they should compare their method with some unsupervised ones at some point.

Thank you for pointing out this. I compared the result obtained by our method with that obtained by the unsupervised method, namely, Louvain clustering (Figure 5C) in the corresponding section. Also, in the section on COVID-19 data, I discussed the comparison between our results and the unsupervised clustering-based result presented in the original paper.

In the introduction, the authors state, "However, these methods do not provide an answer for the appropriate number of clusters and/or an explanation of why some cells are clustered together, but others are separated, leaving uncertainty in the analysis." That point was made throughout the results. However, it would be interesting to reiterate that point in the discussion with a sentence or two.

Thank you for the suggestion. I added relevant points to the Discussion.

The authors could give a longer explanation of the k-NN. What are the features, and how is the smoothing done?

I added details on the Method.

Minor comments: In the paragraph of lines 149-160, figure references are all shifted.

4B=4C, 4C=4D, 4D=4E.

I corrected the errors accordingly.

Figure 3C right is missing an x-label.

Here the x-label is the same as the left panel, as in Figure 3A. Just to make it clear, I moved the label.

Since I cite the sentence in lines 63-65, avoid using "and/or." There is a coma before "but."
Suggestions: Remove the word "very" from line 34. This is a very weak word (irony intended) that contributes nothing to the sentence.

I appreciate the suggestions. I changed them accordingly.

Response to Reviewer #2

Reviewer #2: The author proposes a procedure/method/pipeline composed of multiple steps for the identification and inference of gene regulatory networks, from scRNA-seq. The topic is of relevance since there's a always need for better methods to automate the clustering and analysis of data.

However, the document could be better written, providing a contextualization during each step of the pipeline.
Also, the background could better motivate the need for the tool (with appropriate references) and a better view of the existing tools, justifying the need for another tool.

One example of such is the phrase "There are several methods to explore pivotal elements..." which lacks a reference (if possible a review), especially if the methods are not being mentioned in this document.

Thank you for your suggestion. I added a reference.

Generically, it is a challenging exercise for the reader to understand if this document presents an analysis on specific datasets, or a generic software capable of being applied directly to other data by the wider community.
First and foremost, this should be clarified.

I agree this is important. For clarification, I added a paragraph explaining how the paper is organized at the end of Introduction section.

Phrases such as "Current clustering methods still suffer from uncertainty in determining the appropriate number of clusters" should be avoided, since the proposed method does not address this point also.
This problem is typically addressed by statistical methods, such Silhouette, Elbow, Sum of squares, etc.
The particular method used is not specified.

In page 7 it is mentioned that "By visual inspection of the heatmap of the MDS expression, we determine the number of clusters of clumps as 13". Visual inspection should not be the adequate criteria for a software/pipeline.

Thank you for the comment. I implemented a method using multiscale bootstrap calculation of p-values for clusters and utilizing the result to automate the determination of the appropriate number of clusters. In the method, named BootCellNet2, I added two steps, computation of the unique set of control nodes (while MDS itself is not unique) and the automated determination of the number of clusters. So I believe the point has been addressed now.

Regarding a proper comparison with existing software, it is only performed against SingleR, highlighting a particular case where the proposed method better captures the number of cell populations.
There are many software tools for clustering, GRN inference, and cell type identification using scRNA-seq data.

Thank you for your valuable feedback regarding the comparison of our method with existing software tools.

The primary intention of the analysis presented in the paper was to demonstrate the robustness of our method, particularly given the probabilistic steps involved and the presence of noise within the data. As you pointed out, scRNA-seq data analysis includes a variety of tools for clustering, GRN inference, and cell type identification. However, a direct comparison with all available tools is challenging due to the lack of a universal 'ground truth' in clustering and cell type labeling in scRNA-seq data.

In our study, we chose to compare our method primarily with SingleR, and in this revision, I have added the comparison with unsupervised Louvain clustering. They are well-recognized as starting points for analysis and widely used in the field, thus the comparison will provide a meaningful benchmark.

Additionally, I have performed an expert-based evaluation of the clusters, utilizing my immunology expertise to manually label the clusters. This was done to ensure that despite the probabilistic nature of our method, the clusters identified are not only statistically robust but also biologically meaningful. We believe that this expert validation, combined with the statistical robustness provided by our resampling and automated cluster determination methods is the best we can offer.

We have added these points to the discussion section of our manuscript to better articulate the robustness and biological relevance of our results.

Finally, software-wise, this is a tool/method manuscript. So I really tried to test it, with the following difficulties.
- I managed to install R (despite being 2024).
- I also managed to install all the dependencies/packages to be able to run BoolCellNet, despite:
. dependency management in R is non-existent;
. some of the dependencies being discontinued, forcing me to find the correct old version .tar.gz and installing it manually.
But when finally running it, I got a file not found error since, I guess, some files (and the necessary directory structure) are missing from the github repo.
The software is currently not able to reproduce the results presented in the document, nor is it at a stage to be generically used by the wider community.

I feel sorry for this. I wrote a more detailed explanation on the tutorial, especially how to use it (basically download everything and put them into one folder, though). About installing R and packages, I'm sorry this method is primarily intended to be used with Seurat, since it's widely used.

After getting the feedback, I tried to re-run the program and I happened to find that due to the difference in the version of Seurat, the UMAP plot will be different from what I showed in the current paper. To avoid confusion, I made the following files available from the GitHub respsitory so that you can choose and start the tutorial from any point: RDS files generated by both versions of Seurat, and RData files in which all the results after running the tutorial are saved.

Additionally, I made a Google Forms and put the link for it in the GitHub repository so that one can ask any questions anonymously. If you still have problems, please feel free to ask any questions through the Forms.

Response to Reviewer #3

Reviewer #3: The author presents a pipeline to identify cell types from scRNA-seq data by combining several methods for smoothing, sampling, GRN inference and clustering. The main idea is to increase the robustness of GRN inference by smoothing and resampling of scRNA-seq data and use the obtained GRN to cluster cells. The clustering is performed from the expression of genes being part of a minimal dominating set (MDS) of the GRN. An MDS is a smallest set of genes that influence each other gene in the GRN. The argument is that cell types could be characterized by these genes only.
The pipeline has been implemented in an R script available online, and evaluated on different datasets. It should be noted that the overall approach is not fully automated: for instance, the number of clusters and assignments of cell types involves manual assessment.

As for the last point, The novel procedure BootCellNet2 has been implemented, which involves the computation of unique control nodes as you suggested, and automated determination of the number of clusters. Though assignments of cell types remain manual and expert-based, the method is now automated.

The paper is well written and organized, and figures are clear.
The identification of cellular types and GRN from scRNA-seq data is a very relevant topic, and the proposed methodology seems original and relevant.
Nevertheless, there are a couple of points that could be clarified:
- MDSs are not necessarily unique, which may be worth stating in the paper. It would be insightful to assess the robustness of the classification across equivalent MDSs, and almost-minimal DSs.

Thank you for pointing out this. As I briefly discussed the point in Discussion, although MDS is not unique, we could use critical nodes and intermittent nodes, which appear in all the MDSs or at least in one MDS. I implemented the computation of critical and intermittent nodes, and used them for clustering. The result indicated that the use of the nodes offers more consistent clustering. I named the method BootCellNet2 and added the results in the revised manuscript.

- The validation of the clustering might be strengthened using CITE-seq datasets, by comparing the clustering with BootCellNet against the clustering with ADTs

I agree that this would strengthen the validity of the clustering by the current method. We used CITE-seq data from Stoeckius et al Nat Methods 2017 to perform the validation, as shown in Figure 9. Briefly, the results showed that the clustering by BootCellNet2 offers more accurate results, and complies more with CITE-seq results.

- It is not clear how automated is the cell type label assignment. In the text, this seems a rather expertise-driven labeling. Is part of the cell type identification implemented in the R script?

The labeling itself is expert-based. I wrote this in the Introduction.
