# Supplementary material for: BootCellNet, a resampling-based procedure, promotes unsupervised identification of cell populations via robust inference of gene regulatory networks
Source: PLoS Comput Biol. 2024 Sep 30;20(9):e1012480. doi: 10.1371/journal.pcbi.1012480 (PMC11466406; doi:10.1371/journal.pcbi.1012480)
Supplement: Attachment — Submitted filename: review.docx [file pcbi.1012480.s016.docx]

The work presents a novel method for inference of gene regulatory networks and cell types from single-cell RNA sequencing. The paper relies, among other things, on unsupervised machine-learning techniques such as clustering. However, it solves two major challenges with that kind of approach: inferring the number of clusters and reporting interpretable results.

The authors compare their unsupervised methods with a supervised procedure in Figure 5. However, they mention that there are also unsupervised methods in the literature. They should compare their approach with these, too. As a suggestion, they could contrast their results in the section on hematopoiesis and COVID-19 with the results found by other methods. This would make those sections more pleasant to read. The authors can skip this suggestion, but they should compare their method with some unsupervised ones at some point.

In the introduction, the authors state, "*However, these methods do not provide an answer for the appropriate number of clusters and/or an explanation of why some cells are clustered together, but others are separated, leaving uncertainty in the analysis*." That point was made throughout the results. However, it would be interesting to reiterate that point in the discussion with a sentence or two.

The authors could give a longer explanation of the k-NN. What are the features, and how is the smoothing done?

Minor comments: In the paragraph of lines 149-160, figure references are all shifted. 4B=4C, 4C=4D, 4D=4E.

Figure 3C right is missing an x-label.

Since I cite the sentence in lines 63-65, avoid using "and/or." There is a coma before "but."

Suggestions: Remove the word "very" from line 34. This is a very weak word (irony intended) that contributes nothing to the sentence.
